# Patients’ and nurses’ perception of the Hospice-On-Wheels service in Kazakhstan: a qualitative study

**DOI:** 10.3332/ecancer.2024.1818

**Published:** 2024-12-12

**Authors:** Anele Bekturova, Angelika Saryan, Gulnara Kunirova

**Affiliations:** 1 Asfendiyarov Kazakh National Medical University, Almaty 050000, Kazakhstan; 2 Amazonka Public Fund for Support of Cancer Patients, Taraz 080000, Kazakhstan; 3 Kazakhstan Palliative Care Association (KPCA), Almaty 050020, Kazakhstan

**Keywords:** mobile palliative care teams, Hospice-On-Wheels, palliative and end-of-life care, incurable cancer patients, qualitative research, phenomenology, service design, interviews, qualitative tools and methods

## Abstract

**Background:**

In Kazakhstan, the Comprehensive Cancer Control Plan has led to the establishment of 214 mobile teams funded by the Ministry of Health to offer in-home palliative care for terminal cancer patients. However, due to a shortage of trained and dedicated personnel, only about 25% of these teams provide high-quality, specialised care in the comfort of patient’s homes, with the majority offering only consultative services. Given the country’s sparse population, ensuring dedicated care is challenging and many staff members continue as part-time primary care workers. In this landscape, the ‘Amazonka’ Foundation’s Hospice-On-Wheels initiative emerges as the sole NGO non-government organisation (NGO)-based service delivering in-home palliative care of comparable quality. Distinguished by its contract with the Ministry of Labour and Social Protection, Hospice-On-Wheels enjoys greater organisational flexibility and provides a broader spectrum of services, all while maintaining rigorous standards of multidisciplinary care.

**Aims:**

The aims of this study are to assess the effectiveness and sustainability of an NGO-based in-home palliative care service in Kazakhstan and to discuss the qualitative methodology and its characteristics to Kazakhstani nurses, unfamiliar with quality nursing research.

**Methods:**

The study commenced with a descriptive phenomenological approach and a service design methodology, later transitioning from phenomenological thematic analysis to content analysis.

**Results:**

These rapid research findings highlight the exceptional effectiveness, customer satisfaction and cost-saving advantages of the NGO-based initiative. These benefits extend not only to the government and health authorities but also to patients and their families. The ‘Amazonka’ service significantly enhances the quality of life for incurable cancer patients through improved palliative care. Consequently, expanding their infrastructure and educational initiatives is essential to promote better palliative care in Kazakhstan, particularly in rural areas.

**Discussion:**

The insights from this study can advocate for the home-based palliative care model employed by the ‘Amazonka’ team, encouraging its adoption by both governmental organisations and NGOs in Kazakhstan and beyond, thus opening new opportunities for local funding in resource-limited conditions.

## Purpose and objectives

To examine patients’ and nurses’ perceptions of the local mobile palliative team’s effectiveness, to explore and disseminate the successful experience of the non-government Hospice-On-Wheels initiative in Kazakhstan, to review current literature and international expertise on qualitative nursing research with a focus on the service design method, to propose enhancements to the existing mobile team services in Kazakhstan, to improve the quality of life of incurable cancer patients, to elevate the standards of specialised medical and social support for patients requiring palliative care, to promote greater participation in the palliative care volunteer movement and to educate patients and their families about palliative care options and resources. This study has the potential to serve as an educational tool for nurses and palliative care multidisciplinary teams, facilitating ongoing learning and improvement in palliative patient care practices. By engaging in the qualitative service design process, nurses can develop the skills necessary to conduct their own research projects and educate their colleagues on professional nursing research practices.

## Background

Kazakhstan is the ninth biggest and the largest landlocked country (2.724 million sq km) in the world [1]. With a population of slightly over 19 million and covering an area of 2.7 million sq km, Kazakhstan is characterised by one of the lowest population densities globally, at only 7 people per sq km [2]. This presents significant hurdles in providing accessible health services, particularly in rural areas, where nearly half of the population resides.

In 2023, over 39,000 new cancer cases were recorded, and 127,000 individuals currently live with the oncological diagnosis, including 12,000 children. Despite a decline in mortality rates due to improved cancer care and early detection advancement, around 11,000 cancer patients die from the disease annually [3]. Besides, with a total incidence of 60 tuberculosis cases per 100,000, Kazakhstan is placed among the top 30 countries worldwide with the highest burden of multidrug-resistant tuberculosis [4]. The pressing issue of quality of life for those battling serious non-communicable and communicable diseases, especially at the end of life, has become increasingly urgent, highlighting the need for better palliative care coverage and quality. Kazakhstan is experiencing the early stages of population aging, with distinct demographic patterns across its regions: while the north-east and central areas mirror European trends due to minimal natural population growth and emigration, the southern and western regions witness a rise in birth rates [5]. As the demographic landscape evolves in low- and middle-income countries, the intersection of aging and palliative care needs grows more pronounced [6].

The diverse multicultural character of the country and the presence of various religious beliefs impact approaches toward care for elderly, sick or dying individuals. Traditionally, elderly and dying people are cared for by their family members in homes, while hospices, as inpatient medical facilities, are largely stigmatised due to negative association with death and dying, as well as general belief surrounding hospice care as something that represents ‘abandonment’ on the part of the family. Providing end-of-life care at home, however, creates a serious financial burden for families.

Acknowledging the impending challenges, Kazakhstan addresses the surge in chronic non-communicable diseases, shifting demographics towards aging, healthcare access disparities between urban and rural areas and regional cultural nuances. Recognising the urgency, the nation prioritises policy and systemic interventions to strengthen long-term and palliative care infrastructure, aiming for comprehensive support for its aging and terminally ill population, fostering high-quality, inclusive, accessible and affordable palliative care.

### Palliative care and policymaking

According to the second edition of the Global Atlas of Palliative Care, there are presently 107,430 individuals in Kazakhstan who require palliative care, including 4,900 children [7].

Efforts have been made recently to enhance palliative care services in Kazakhstan. Although there is no officially documented national strategy for palliative care, the country’s stance is reflected in various legislative measures. Notably, in 2020, the Code on People’s Health and Healthcare System acknowledged palliative care as an essential form of medical care, dedicating a specific chapter to its provision [8]. The delivery of palliative care is governed by a national Standard sanctioned by the Ministry of Health [9]. Furthermore, the Comprehensive Plan on Cancer Control for 2018–2022, as well as the subsequent iteration covering 2023–2025, has a section dedicated to palliative care, which provides for the establishment of a nationwide network of multidisciplinary mobile teams for terminal cancer patients, in addition to 14 inpatient palliative care departments [10, 11].

Palliative care to both cancer and non-cancer patients is also provided in dedicated hospices or palliative care centers. The total number of beds of over 2,000 aligns with the established standard of 10 beds per 100,000 population [9]. However, about 38.5% of these beds represent isolated beds or wards in local (district) hospitals, where personnel are not fully prepared to provide quality multidisciplinary care to patients with complex palliative care needs.

On a primary care level, patients with severe incurable diseases receive medical support from their family doctor (at least once a month) and family nurse (at least once a week), highlighting a commitment for primary care teams to offer palliative care within the national health system.

Inpatient, home-based palliative care, as well as 19 essential medicines, including 3 strong opioids are provided free of charge, as part of the State-guaranteed Package of Free Medical Care [12].

In Kazakhstan, there are 11 clinical protocols specifically on palliative care, including one on pain management. These were originally approved in 2013 and are regularly reviewed.

Additionally, in 2022, roadmaps were formulated for both adult and pediatric palliative care, aiming to chart the course for the advancement of palliative care services, improvement of a legal base, access to opioids, education and training [13, 14].

### In-home palliative care and role of NGOs

In-home palliative care services in the form of mobile teams were originally established by three non-government organisations (NGOs) in Karaganda, Almaty and Taraz. While the Karaganda team had to terminate its activities due to the lack of grant funding, it is remarkable how the Almaty team evolved from its roots in a non-profit organisation to being integrated into the state-budgeted branch of the oncology service.

There was a long process of raising concerns and understanding at the government level regarding the efficiency of home-based palliative care in terms of universal coverage, healthcare economics, family satisfaction and social justice as ‘the lack of sufficient state funding and universal health coverage results in high out-of-pocket medical expenses, which consume a large portion of family income’ [15]. As a result, based on the Almaty model, the introduction of mobile teams as part of the Cancer Control Plan in 2018 marked a significant step forward in providing palliative care services. To date, 214 mobile teams are functioning nation-wide, but only 40% of terminal cancer patients are covered by palliative mobile home assistance [16]. The fact that only 25% of physicians, nurses, social workers and psychologists, working in these mobile teams, received appropriate training in palliative care highlights a significant gap in the system [16]. This lack of dedicated personnel and specialised training leads to disparities in the quality and scope of care provided to patients.

Mobile teams play a vital role in reaching patients in remote areas or those who face barriers to accessing inpatient facilities or specialised hospices. By bringing palliative care directly to patients’ homes, these teams are aimed at addressing physical symptoms and providing much-needed psychological, social and spiritual support to patients and their families.

**Figure 1.**
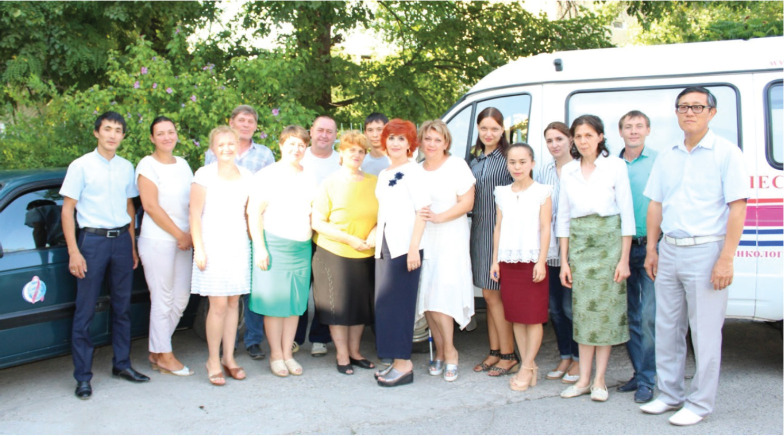
The multidisciplinary team of Hospice-On-Wheels.

Hospice-On-Wheels, founded in 2011 by the ‘Amazonka’ Foundation, is a non-government project, which receives financing from the local social protection budget and charity (Figure 1). ‘Amazonka’ remains the sole NGO-based palliative mobile team of its kind in the country, which provides organisational flexibility and enables a wider range of services to be offered while maintaining a high standard of multidisciplinary palliative care.

Established in 2013 through the initiative of the aforementioned NGOs, the Kazakhstan Palliative Care Association (KPCA) has evolved into a pivotal entity, serving as an umbrella organisation for NGOs, hospices and various palliative care programs and services in Kazakhstan. With a primary focus on advocacy, legislation improvement, education and training, government engagement, awareness campaigns and fostering volunteerism, KPCA has emerged as a driving force in the advancement of palliative care across the nation, ‘playing a leading and connecting role in promoting the humanistic philosophy and efficient practices of palliative care in Kazakhstan’.

Over the past decade, KPCA has garnered significant recognition and respect within Kazakhstan and beyond, establishing networks and collaborations worldwide. In a testament to its credibility, in 2022, the President of KPCA was appointed as the Chief External Expert in Palliative Care for the Ministry of Health of the Republic of Kazakhstan [18].

### ‘Amazonka’ Hospice-On-Wheels project

**Figure 2.**
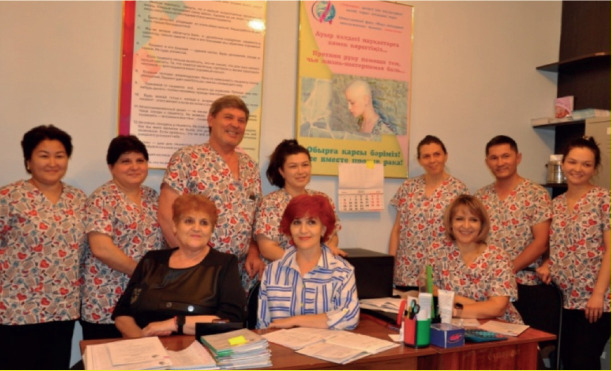
Angelika Saryan (in the center) and ‘Amazonka’ Foundation team.

The Hospice-On-Wheels (Figure 2) stands out as the only active and steadfast NGO-operated in-home palliative care service for adults, not only in Kazakhstan, but also in Central Asia. Founded on December 6, 2011, in Taraz, the Public Foundation ‘Fund for the Support of Cancer Patients’ Amazonka bears the imprint of Angelika Saryan’s remarkable journey. Angelika, the founder of ‘Amazonka’, a dedicated healthcare provider and psychologist, is also a cancer survivor who has undergone nearly 100 chemotherapy sessions. Her own battle with cancer began in 2006, and in 2007, she narrowly escaped death due to medical negligence and individual response to the chemotherapy [19]. Following her recovery, Angelika Saryan committed herself to aiding palliative cancer patients and their families. The ‘Amazonka’ organisation, inspired by the legendary female warriors known for their courage and the removal of one breast to wield a longbow effectively, was founded to support women fighting breast cancer, embodying the strength and resilience of these mythical figures in their mission to empower and assist those facing the challenges of the disease [20].

For several years, Hospice-On-Wheels has operated under an independent contract with the Department of Social Protection, while also garnering support from private sponsors and charities. This support structure enables ‘Amazonka’ to offer a diverse array of services and provisions to incurable cancer patients and their families, entirely free of charge.

The organisation is composed of a dedicated team of 21 staff members, comprising its leader, accountant, lawyer, receptionist, manager, two doctors, psychologists, care aides, drivers and three nurses and social workers. Additionally, there are 22 regular volunteers, including a pastor and a mullah, actively involved in the project [20].

To facilitate their palliative operations, ‘Amazonka’ maintains two medically equipped cars, enabling the team to divide into two mobile groups and cover all residents of Taraz – the fifth biggest city in Kazakhstan.

Typically, all team members adhere to regular working hours from Monday through Friday. However, they demonstrate flexibility, often extending their work hours into evenings, weekends and holidays when necessary. In critical situations, the team remains available either in person or reachable by phone for immediate assistance.

The organisation’s mission is succinctly encapsulated in the phrase ‘lending a helping hand to anyone whose life is an unbearable pain’ [20]. This ethos guides their efforts in providing compassionate support to those in dire need. Aligned with this mission, the organisation has set forth several goals:

- Delivering free and accessible palliative care to terminally ill cancer patients within the comfort of their homes. This care is administered by a multidisciplinary team comprising physicians, nurses, psychologists, social workers and spiritual counselors.
- Enhancing the quality of life and ensuring dignity during the final days of terminal patients, aiming to alleviate suffering and provide holistic support.
- Facilitating transportation for palliative patients to hospitals or specialist appointments via medically equipped minivans, ensuring they receive necessary medical attention and support.
- Conducting training sessions for new healthcare providers, volunteers, patients and their families to empower them with the necessary skills and knowledge to navigate the challenges of terminal illness.
- Increasing awareness and disseminating vital information within the community regarding palliative care, end-of-life issues and available support resources.
- Providing essential medical equipment and supplies, including medical beds, walkers, oxygen tanks, wheelchairs, pressure-relief mattresses, portable bathtubs, bedpans, medications, diapers and more, to alleviate discomfort and enhance comfort at home.
- Offering personalised consultations, whether in-person or online, encompassing medical, legal, psychological, spiritual or social aspects, tailored to meet the unique needs of patients and their families.
- Assisting in hospital admission when necessary, ensuring seamless transitions and continuity of care for patients requiring specialised medical interventions (Figure 1).

### Project purpose and objectives

The purpose of this research is multifaceted, focusing on the exploration and assessment of the sustainability and effectiveness of mobile palliative care teams in rural areas with low population density. Specifically, it examines the ‘Amazonka’ team’s decade-long successful operation from the perspectives of both patients and nurses. The objectives are comprehensive, aiming to:

1. Investigate and assess: Examine the factors contributing to the success of the ‘Amazonka’ hospice-on-wheels initiative and assess the in-home palliative care model in rural areas.
2. Literature review and educational development: Review existing literature and international expertise in qualitative nursing research, particularly through service design, and use the study as an educational base for independent quality research by other mobile teams or magistrate nurses.
3. Quality of life improvement: Enhance the quality of life for individuals with incurable conditions and improve the delivery of quality medical and social in-home services for palliative care patients and their families.
4. Community engagement and awareness: Promote participation in the palliative care volunteer movement, raise community awareness about palliative care and educate patients and their families on palliative care services.
5. Skill development: Equip family caregivers and other palliative care providers with effective caregiving skills.

## Methods

In developed nations, qualitative nursing research methodologies are a staple within the nursing discipline, yet in Kazakhstan, there is a discernible deficit in both the knowledge and application of such research among nurses. Traditionally, the nursing role within the country has been confined to ancillary support for physicians, with minimal encouragement or avenues for engaging in autonomous research endeavors. Contemporary efforts are underway to amend this historical oversight; however, these initiatives are impeded by the constraints inherent to a low-income nation. These include a limited pool of nurses with tertiary education, a dearth of scholarly nurse-educators proficient in research methodologies and an underdeveloped healthcare infrastructure, with palliative care being notably affected [21]. Introducing qualitative inquiry to nurses is crucial, given its rarity among medical professionals in Kazakhstan and Central Asia, where quantitative research predominates.

In our study, we opted for qualitative methodologies, specifically a descriptive phenomenological study. This approach is grounded in ‘qualitative evidence’, which encompasses narrative, reflective or anecdotal information that necessitates discernment to decode the data concerning the human experience [22]. Additionally, qualitative research delves into ‘human responses within a given situation and context, as well as the significance individuals attribute to those circumstances’ [23], making it particularly relevant for vulnerable individuals and mobile palliative care services.

The service design methodology involves the strategic arrangement and management of business or healthcare resources, such as personnel, equipment and procedures. This is done with the dual objectives of: 1) enhancing the experience of medical staff and 2) consequently, bettering the experience of palliative care patients [25]. It is essential to conceive of the service as a series of connected activities that reflect the lived experiences of those involved, thereby establishing authenticity [24].

In the course of our investigation, we initially employed a descriptive phenomenological study in conjunction with service design. However, as the research progressed, our approach transitioned from a phenomenological thematic analysis to a content analysis. This shift allowed us to deconstruct the interview data and reassemble it into coherent descriptions and interpretations [22].

### Data gathering

The data-gathering process was expedited and completed within a 2-month span, specifically during October and November of 2023. We enlisted participants from the staff of ‘Amazonka’ and their 103 patients who were under care at the time. The study included semi-structured interviews with two nurses, one psychologist and four patients, each lasting between 45 to 120 minutes. These interviews were conducted in Russian in the city of Taraz. All interviews were meticulously monitored, audio-recorded and transcribed for subsequent analysis. The key informants were prompted with ten open-ended questions regarding their personal experiences and emotions (Tables 1 and 2).

### Stages of service design

**Figure 3.**
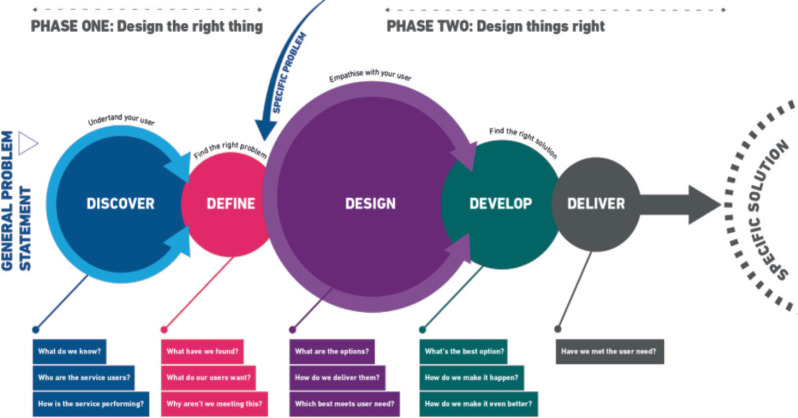
Stages of service design adopted from Google internet.

The service design helped to organise and systematise the qualitative research. Using the four stages (Figure 3) and assigned tools and instruments for each stage, minimised the workload and assisted with efficient data collection before, during and after semi-structured in-depth interviews that were gathered through this research [23].

**Figure 4.**
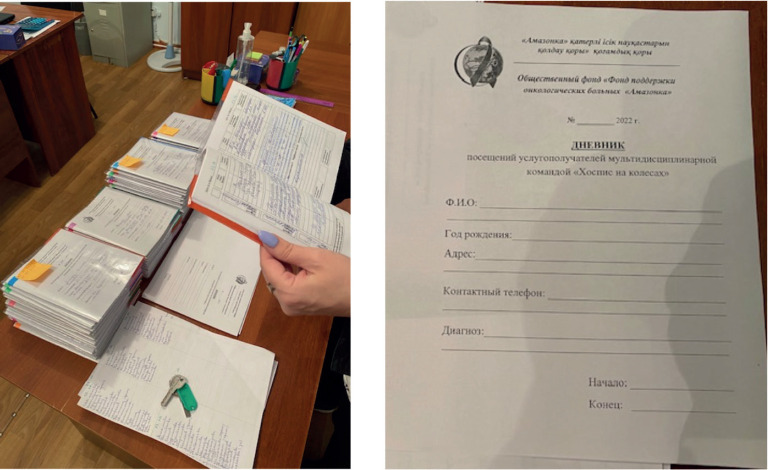
Amazonka’s innovative tools.

During the first stage of the study (Define stage), we explored what was in place and searched for issues relevant to palliative care provision in remote areas and in-home services. The original plan of using evaluation tools, such as the discovery of project goals, guided observation tools and ‘Amazonka’ innovative tools (Figure 4) as an evaluation base for discussions during the interviews and observations, was used [23]. ‘Amazonka’ team created their own visual tool that helped them to assess their patients: they used regular school diaries (Figure 4) as monitoring log journals.

The ‘Amazonka’ team created their own visual tool that helped them to assess their patients. They used regular school diaries (Figure 4) as monitoring log journals.

During the discovery stage, we composed ten questions to cast a wider net as a quality researcher, Sandelowski, suggested to discover new unexpected outcomes [22]. However, when the study progressed, we narrowed and directed it to the three most critical questions for our two groups’ participants. For nurses and other medical providers, we elaborated more on the last two questions (Table 1), whereas for the palliative patients, we concentrated on questions five, seven and ten (Table 2).

**Figure 5.**
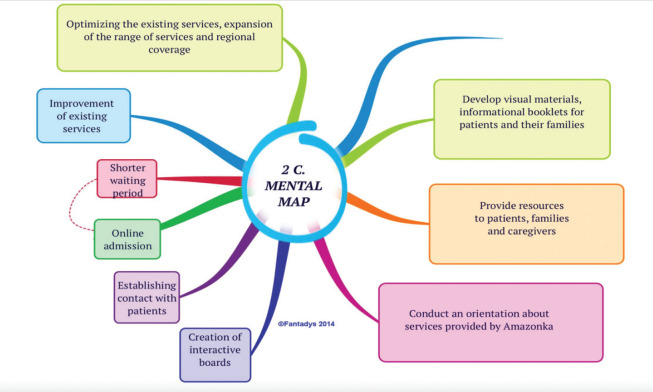
Mental map.

At the second stage (Discover), we conducted semi-structured interviews, continued thorough observation and started analysing collected data. We used empathy and mental maps (Figure 5) as organising tools. Empathy maps helped us to analyse each individual and highlight repeated themes, and mental maps were used to point out key insights.

At the third stage (Develop), medical participants described their typical patients, created their personality cards and explored their needs, problems and life credo.

Together, with active contributions from all workers from ‘Amazonka’: psychologists, social workers, doctors and nurses, we established four typical personality profiles. In order to do that, we used Mayers-Briggs 16 personality profiles that we limited to only two comparable classifications. The first one was extroversion versus introversion, where we evaluate how people restore their energy and the second category was thinking versus feelings, on how people make their decisions [25]. At the end, we came out with four distinctive profiles:

1. 68-year-old female patient with brain cancer and glioblastoma. She is sangvinic, ambivert, kind, emotionally stable and pragmatic. Her credo is ‘you are driving me crazy;, considered this phrase responsible for developing oncology. Her needs and requests are motivation to recovery from cancer, adaptive to her illness, configurates herself to be relax, stable and read books. Her issues are slowness, anxiety, speech impairment and acceptance of her cancer stage. Her abilities are mood with + positive shade, self-improvement, believe in God, helping kids and optimism.
2. 29-year-old male patient with a brain tumor. He is phlegmatic, emotionally stable, kind, pragmatic, reasonable and calm. His needs and requests are motivation for the recovery process, adaptive to his cancer and hope for a recovery. He likes to read spiritual books. His issues are slowness, apathy, anxiety and depression. His abilities are mood with + positive shade, self-improvement, optimism, pragmatic and believe in God.
3. 64 years old male patient with lung cancer. He is melancholic, introvert, total pessimism, kind, loving, self-dedication and thoughts of death. His needs are acceptance of his illness without any hope, perfectionism towards himself, absence of motivation, depletion of the psycho-biological reserve and depressed emotions. His issues are depression, irritability, self-isolation, fear and alienation. His abilities are mood with - negative shade, self-improvement, pragmatic, resistant to contacts, negativism, but believe in God.
4. 35-year-old female patient with an ovary tumor. She is choleric, irritability with defensive aggressiveness and stalling on denial of her illness. Her needs and requests are to decrease pain level and intoxication, increase emotional stability, mood control and availability of internet. Her issues are irritability, denial of her tumor, self-sabotage during remission periods, accusation of others even the medical staff, rapid hysterical mood fluctuations and not trusting others. Her abilities are optimism, hope for a recovery and stubbornness.

At the fifth stage, we looked at all of the possible solutions to the problems identified during the define stage. As part of our commissioning process, we monitored all potential options for service delivery, not just how the services have been delivered. This stage’s objectives were to identify various solutions for meeting our customer’s needs; to ensure the user is still at the heart of what we are doing; to be able to compare and evaluate the different delivery options. The delivery phase is also a point for feedback lessons from colleagues and palliative patients, sharing new knowledge, lived experiences, insight tools or ways of working [22]. Finally, the writers would continue with the fourth delivery stage in 12 months period. Recommendations for other mobile palliative teams and other involved stakeholders were also developed at this stage.

As a result, we searched, interpreted, reflected and extracted repeated codes/labels and insights, as well as reoccurring themes and patterns out of these five selected questions about participants’ lived experiences (Figure 5).

## Discussion

### Integration and implementation

Usually, qualitative studies require prolonged time as evidence could evolve and hidden issues or problems could be revealed. Also, in-depth qualitative analyses repeated after some time could disclose new perspectives and points. ‘Qualitative studies use an emergent design that takes shape as researchers make ongoing decisions reflecting what they have already learned’ [22]. The recommended time frame is 6–12 months. However, we experimentally limited ourselves with only 2 months period for our interviews’ period and performed this study as a qualitative ‘blitzkrieg’.

### Sample and sampling strategies

The difference between quantitative and qualitative research is huge in the selected sample quantity. In our qualitative study, the sample is quite small and consists of one psychologist out of two, two nurses out of three and four patients out of 103. We thoroughly work on our selection sample, especially in our patient group to cover representative personalities. We were led by the assumption that we could get different data from diverse personality types of participants. This assumption was supported at the beginning of our research as we faced a challenge to recruit volunteers from all four selected groups of palliative patients.

Out of those four typical personality profiles, our team easily recruited volunteers from the first group, the volunteers from the second and fourth groups were engaged with less ease, and the hard time was finding a willing sample from the third group. However, all volunteers, who agreed to participate in our in-depth interviews, signed their informed consent; were eager to share and describe their lived experiences and feelings; and cordially expressed their gratitude of being helped by hospice-on-wheels ‘Amazonka’.

As the criterion for determining the sample size, we used data saturation that was achieved by producing ‘a richly textured picture’ of the participants’ lived experience [22]. Despite the abovementioned assumption, we were able to reach data saturation by interviewing four patients from four personality profiles.

During thorough interviews, a number of themes and the data saturation key insights emerged (Figure 5). The main themes were about the difficult experience and the long way from the earlier diagnostic of cancer, acceptance of it, and connection with the ‘Amazonka’ team. There were repeated themes of being left alone, being ‘one on one’, untouchable fears, unmet needs and prolonged suffering. However, the current situation with even progressing illness, but being supported by mobile palliative team, reveal positive insights, such as hopefulness, relieve and gratitude.

Most codes, subcategories and categories were evolved around the well-known concept of ‘total pain’ and ‘total suffering’ by Dame Cicely Saunders [26] and five stages of illness acceptance described by Swiss psychiatrist Elisabeth Kübler-Ross [27]. For example, all interviewed participants from the different groups shared their lived experiences of shock, numbness, denial, emotional swings, envy, fearfulness,

disorientation, guilt, panic, loneliness, isolation, negotiation, depression, reorganisation, acceptance and finally adaptation. The lived perceptions changed slightly for different personality types, but the time frame of the shared experiences varied for all participants. Similar codes and insights were discovered in relation to the concept of total pain. Almost all palliative patient mentioned physical changes such as weakness, inability to sleep or cognitive changes; psychological transformations such as isolation and loneliness; social alterations such as fear, position changes and inability to control situations; and spiritual adjustments with their religion, values and meaning of life.

Finally, many participants shared and suggested very insightful ideas about what they would love to get or need through the support of the mobile palliative team, hospice-on-wheels, which were summarised in the form of the mind map in Figure 5.

### Quality standards and scientific rigor

Besides finding an existing practice gap in the provision of palliative care in Kazakhstan and searching the academic studies for existing supportive evidence, the researcher should consider and promote the sustainability of this new innovation. In order to do so, they should be cognizant of three pillars for any change adaptation and maintenance: the abilities of stakeholders, institutes and the wholeness of healthcare systems/infrastructures [28]. Institutionalisation or sustainability of project implementations could be achieved by spreading or scaling up horizontally or vertically. As these are continuously evolving processes, they could not be static and could be dynamically changing in any direction from relapsing to an old status quo, to a developing adaptation and routinisation of a proposed innovation.

### Study limitations

Quality studies require extended time periods for data collecting and analysis due to evolving design and intensive analysis of multiple lived realities of participants. However, we purposefully limited our study to only 2 months and could miss more key insights. Another aspect was related to sample and sampling strategies as we were recruiting voluntary participants, we probably missed a particular category of palliative patients with specific personality profiles or being so sick that they could not participate in our research.

### Ethical considerations. Informed consent

In the progression of our project overall, we were guided by the four basic ethical principles of: beneficence, justice and respect for individuals and communities [29]. In order to follow these broad ethical principles through our project, we protected our stakeholders’ rights for privacy (justice), we produced as much benefits as possible for participants (beneficence), and we created an open trustful environment through our interviews and observations with complete disclosure and voluntary participation (respect) [30]. Also, we informed the participants that their expressed opinions and points of view during interviews are anonymous and confidential. All interviews and observations were conducted with permission and signed consent from all stakeholders, including patients, their family members and nursing and health professional teams’ members.

## Conclusion

### Strengthening infrastructure and capacity

Studying, supporting and expanding the local non-government palliative mobile teams, like ‘Amazonka’ can lead to long-term cost-savings benefits as their enthusiastic contribution decreasing the load from hospitals, other medical health professionals and organisations, as well as from palliative patients and their families. Promoting understanding and raising awareness about the palliative mobile teams in government structures would improve supportive policymaking and creating ‘a policy umbrella’ over NGOs, providing palliative care. Moreover, according to the palliative care expert review, ‘this research has given a shot to introduce a creative and cultural-based intervention’ to end-of-life care in Kazakhstan.

### Educational strategies

Given the scarcity of qualitative research in nursing within Kazakhstan, there is a pressing need to incorporate and prioritise palliative care education within medical and nursing training programs. The absence of formal recognition for palliative care specialists underscores the urgency of this educational reform. In view of the fact that ‘palliative care is not included as a mandatory subject in medical or nursing curricula in Kazakhstan, and there is no such specialty as palliative care physician or nurse’ [15], and the limited number of nursing researchers, it is relevant to introduce, educate and mentor more Kazakh nurses to qualitative nursing research, particularly the visual application of service design. By adopting and tailoring service design methodologies, we can enhance the palliative care landscape. The commendable work of the ‘Amazonka’ teams serves as a testament to the potential impact of such initiatives and stands as a model worthy of emulation, both in Kazakhstan and in other Central Asian countries.

## Conflicts of interest

The authors declare that there are no conflicts of interest.

## Funding

No funds were received for this study.

## Author contributions

AB designed and performed the qualitative study, collected and analysed the data, wrote and composed the paper. GK provided and reviewed data and wrote the article. AS conceived and designed the research, collected and contributed data and consulted on the paper. All authors contributed to the paper and approved the submitted version.

## Figures and Tables

**Table 1.** Ten questions for nurses to investigate ‘what and why’.

1. What is your name?
2. What is your general professional experience?
3. Duration of work in the organisation
4. What challenges did you face in your organisation?
5. Describe your patients
6. Describe problems that they face
7. Describe the admission process and what difficulties patients experience at admission
8. Did you have any conflict situations in your organisation or with patients and their families?
9. What needs and can be improved in your work?
10. What recommendations and suggestions would you make to be implemented in the future?

**Table 2.** Ten questions for patients to explore their perspectives.

1. The main purpose of your appeal?
2. How did you find out about us and who referred you?
3. What difficulties did you experience before contacting us?
4. Are you satisfied with the services provided by the mobile team?
5. How long did it take for your disease to develop to the state you are in now?
6. What kind of help did you receive before us?
7. What would you suggest to improve in our work to take a better care of you?
8. Are you satisfied with all the team’s services, and which ones do not satisfy with?
9. What would you like to improve in our organisation process?
10. What other suggestions and wishes do you have?
